# Sphingosine 1-Phosphate Receptors and Metabolic Enzymes as Druggable Targets for Brain Diseases

**DOI:** 10.3389/fphar.2019.00807

**Published:** 2019-07-23

**Authors:** Sara Grassi, Laura Mauri, Simona Prioni, Livia Cabitta, Sandro Sonnino, Alessandro Prinetti, Paola Giussani

**Affiliations:** Department of Medical Biotechnology and Translational Medicine, University of Milan, Milan, Italy

**Keywords:** sphingosine 1-phosphate, fingolimod, FTY720, sphingosine 1-phosphate receptors, sphingosine kinase, sphingosine 1-phosphate phosphatase, sphingosine 1-phosphate lyase

## Abstract

The central nervous system is characterized by a high content of sphingolipids and by a high diversity in terms of different structures. Stage- and cell-specific sphingolipid metabolism and expression are crucial for brain development and maintenance toward adult age. On the other hand, deep dysregulation of sphingolipid metabolism, leading to altered sphingolipid pattern, is associated with the majority of neurological and neurodegenerative diseases, even those totally lacking a common etiological background. Thus, sphingolipid metabolism has always been regarded as a promising pharmacological target for the treatment of brain disorders. However, any therapeutic hypothesis applied to complex amphipathic sphingolipids, components of cellular membranes, has so far failed probably because of the high regional complexity and specificity of the different biological roles of these structures. Simpler sphingosine-based lipids, including ceramide and sphingosine 1-phosphate, are important regulators of brain homeostasis, and, thanks to the relative simplicity of their metabolic network, they seem a feasible druggable target for the treatment of brain diseases. The enzymes involved in the control of the levels of bioactive sphingoids, as well as the receptors engaged by these molecules, have increasingly allured pharmacologists and clinicians, and eventually fingolimod, a functional antagonist of sphingosine 1-phosphate receptors with immunomodulatory properties, was approved for the therapy of relapsing–remitting multiple sclerosis. Considering the importance of neuroinflammation in many other brain diseases, we would expect an extension of the use of such analogs for the treatment of other ailments in the future. Nevertheless, many aspects other than neuroinflammation are regulated by bioactive sphingoids in healthy brain and dysregulated in brain disease. In this review, we are addressing the multifaceted possibility to address the metabolism and biology of bioactive sphingosine 1-phosphate as novel targets for the development of therapeutic paradigms and the discovery of new drugs.

## Introduction

**Figure 1:**
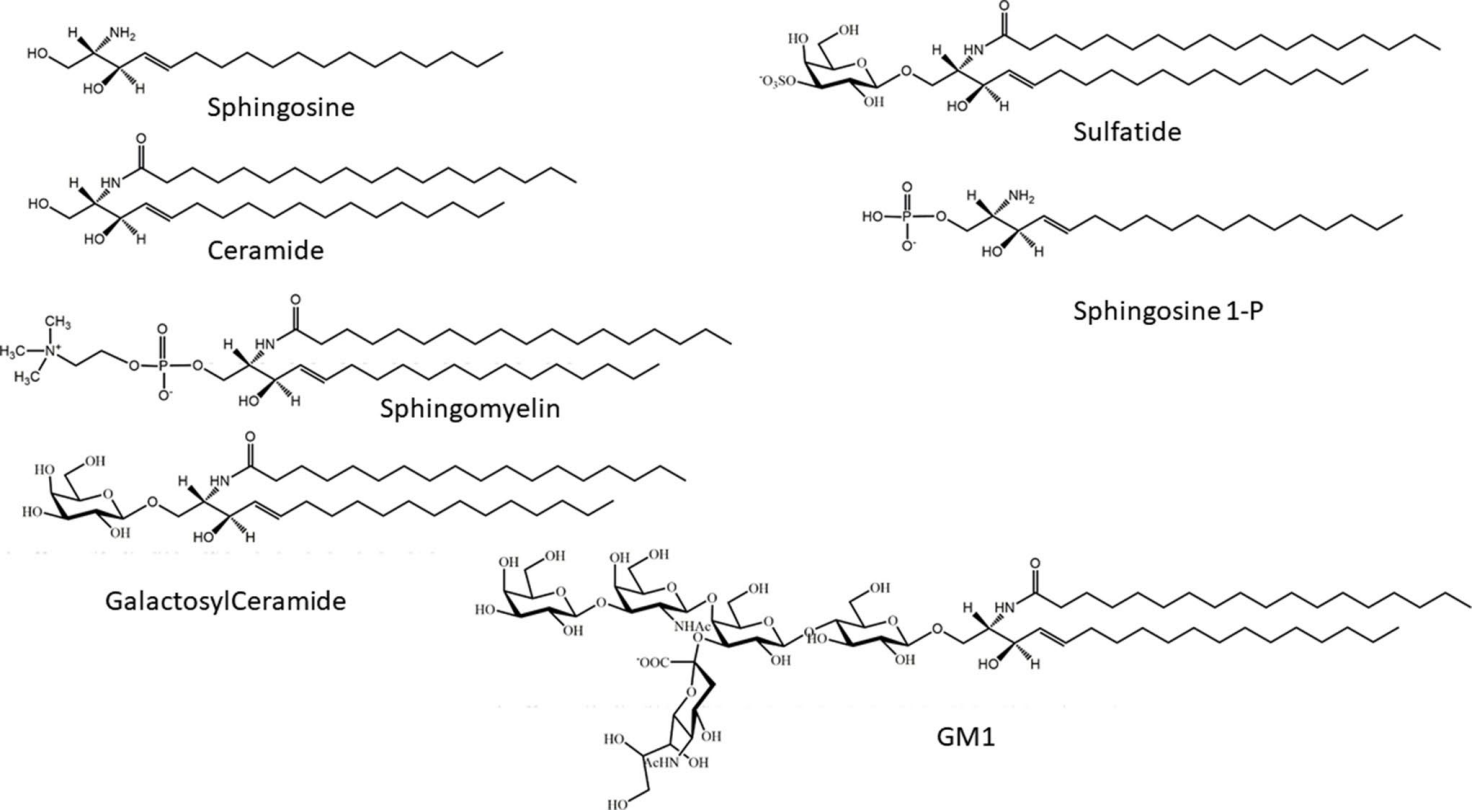
Chemical structures of sphingosine, ceramide, sphingosine 1-phosphate, galactosylceramide, sphingomyelin, and monosialoganglioside GM1.

Sphingolipids are a wide group of eukaryotic cellular lipids, whose common structural feature is the presence of a 2-amino-1,3-dihydroxy-octadec-4-ene, trivially known as “sphingosine” (Roisen et al., 1981) (2*S*,3*R*,4*E*). In addition to the C18 molecular species, which is usually the most abundant in complex sphingolipids in mammals, structures with shorter and longer alkyl chains have been identified as minor components in different biological samples, and complex glycosphingolipids containing sphingosine with 20 carbon atoms are relatively abundant in mammalian brain and in cultured neurons (Sonnino and Chigorno, 2000). Usually, the term “sphingolipids” is referred to as the complex amphipathic lipids of this family, whose hydrophobic moiety is represented by ceramide. In ceramide, the sphingosine is linked *via* an amide bond with a fatty acid, whose structure can greatly vary for its acyl chain length and the presence of double bonds. Ceramide is the precursor for the biosynthesis of amphipathic sphingolipids, characterized by the presence of a polar head group of different nature and chemical complexity linked to the hydroxyl group at the position 1 of ceramide. Phosphocholine is the hydrophilic head group of sphingomyelin (SM) (the only membrane phosphosphingolipid present in mammals). On the other hand, a phosphate group linked to the hydroxyl group is present in sphingosine 1-phosphate and ceramide-1-phosphate. In glycosphingolipids, the polar head group is represented by a single sugar (in cerebrosides) or by an oligosaccharide chain that can be relatively complex, as in polysialogangliosides. In addition to glycosphingolipids containing neutral sugars, two families of glycosphingolipids in eukaryotes are characterized by the presence of acidic residues in the oligosaccharide chain: sulfatides, containing one or more sulfate groups, bond *via* *O*-glycosidic linkage on a glucose or galactose residue; and gangliosides, hallmarked by the presence of one or more sialic acids, a family of sugars characterized by the presence of a carboxyl group (Schauer, 1982) (**Figure 1**).

Sphingolipids are in general minor cell membrane components. Most glycosphingolipids do not form bilayers spontaneously in aqueous environment; however, they can accommodate in the glycerophospholipid bilayer, thanks to their hydrophobic moiety, ceramide. Sphingolipids are ubiquitous components of mammalian cell membranes; however, their distribution is highly organ- and tissue-specific. Indeed, in some cases, imaging mass spectrometry is also revealing a cell-specific distribution. For example, neuron subpopulations located in very close proximity can bear dramatic differences in their sphingolipid repertoire (Hirano-Sakamaki et al., 2015; Sugiyama and Setou, 2018). The nervous system is the mammalian tissue with the highest concentration in sphingolipids. Within the nervous system, SM is highly expressed in oligodendrocytes and in neurons. Galactosylceramide and its sulfated derivative, 3-*O*-sulfogalactosyceramide (sulfatide) are highly expressed in myelin, whereas polysialogangliosides of the ganglio series are abundant in neurons (we showed that ganglio series gangliosides represent about 5% of total amphipathic lipids in cultured cerebellar granule cells; Prinetti et al., 2000). Even if minor components in average cell membranes, sphingolipids are highly concentrated in the plasma membrane and asymmetrically distributed in the exoplasmic leaflet. Moreover, sphingolipids are not homogeneously distributed at the cell surface, but they tend to cluster, forming sphingolipid-rich membrane areas or “domains” (Sonnino et al., 2007; Sonnino et al., 2014); thus, their local concentration within a specific membrane microenvironment can be very high. Plasma membrane sphingolipids, and in particular glycosphingolipids (Sonnino and Prinetti, 2010), do exert important biological functions, affecting the properties of plasma membrane-associated proteins (e.g., growth factor receptors, adhesion molecules) *via* direct interactions or *via* the modulation of the protein membrane microenvironment (Loberto et al., 2005; Rivaroli et al., 2007). Glycosphingolipid-mediated interactions can also involve ligands occurring on adjacent cells (*trans* interactions). For example, the *trans* interaction between sulfatide and galactosylceramide clustered in distinct membrane microdomains at the juxtaposed membranes of myelin wrap plays a relevant role in the proper maintenance of mature myelin function, as well as in myelin formation (Boggs et al., 2010). On the axonal side, the polysialogangliosides GD1a and GT1b organized in clusters do engage *trans* interactions with the glycoprotein MAG on the innermost myelin sheath (Schnaar and Lopez, 2009), contributing to long-term axon–myelin stability.

**Figure 2:**
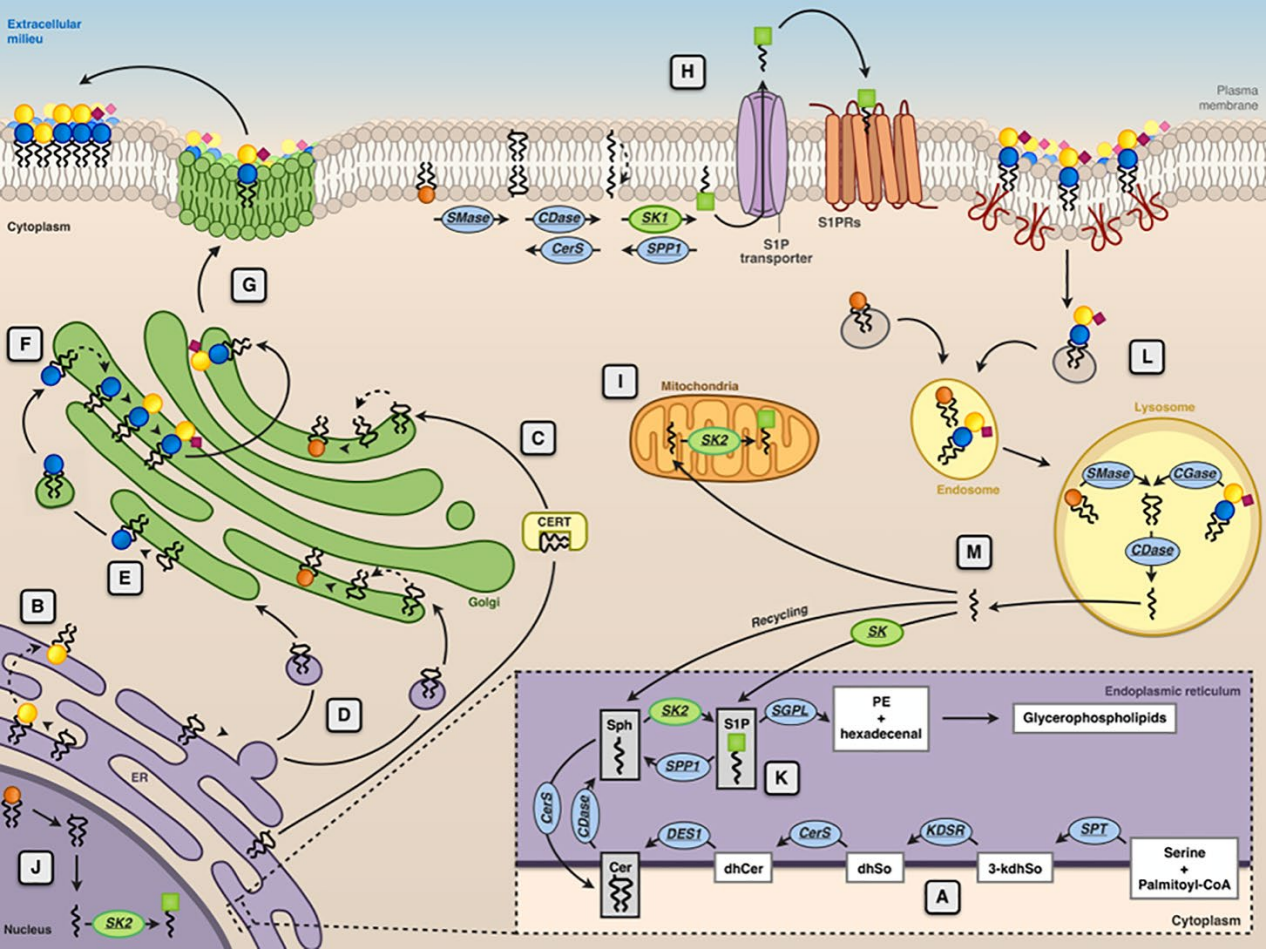
Subcellular compartmentalization of sphingolipid metabolism. The endoplasmic reticulum (ER) is the subcellular site where the *de novo* ceramide (Cer) synthesis occurs **(A)**. Here, the synthesis of the sphingoid bases (sphingosine) starts with the condensation of palmitoyl-CoA and serine, catalyzed by serine palmitoyltransferase (SPT). The product of this reaction is 3-ketodihydrosphingosine (3-kdhSo), which is later reduced to dihydrosphingosine (dhSo) *via* the action of 3-ketodihydrosphingosine reductase (KDSR). Then, dhSo is acylated generating dihydroceramide (dhCer). In humans, this reaction occurs through the activities of six different ceramide synthases (CerS). Dihydroceramide is then unsaturated to ceramide by the enzyme dihydroceramide desaturase 1 (DES1). At ER level, Cer is either transformed into GalCer (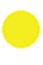) by CGalT **(B)** or delivered by ceramide transport protein (CERT) **(C)** or through vesicular transport **(D)** to the Golgi apparatus for the synthesis of sphingomyelin (SM 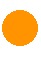) by SMS1 and glucosylceramide (GlcCer 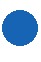) by GCS **(E)**. At the Golgi, GlcCer is transformed into lactosylceramide (LacCer 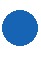

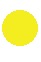
) and complex GSLs (e.g., GM3 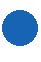

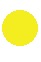

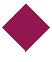
) **(F)**, which are then delivered to the plasma membrane *via* Golgi vesicular transport **(G)**. At the plasma membrane level, SM can be converted into ceramide by sphingomyelinase (SMase), ceramide can then be transformed into sphingosine (Sph) by ceramidase (CDase), and sphingosine is converted into sphingosine 1-phosphate (S1P 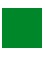) by sphingosine kinases (SK1) **(H)**. S1P is then transported across the membrane by specific transporters. Phosphorylation of sphingosine yielding to S1P because of the action of sphingosine kinase 2 (SK2) can occur at the mitochondria **(I)**, nucleus **(J)**, and ER **(K)**. In the ER, S1P can be either irreversibly cleaved by S1P lyase or dephosphorylated back to sphingosine by a specific phosphatase (SPP1). Membrane sphingolipids are internalized *via* caveolae-dependent endocytosis and, in the lysosome, they are degraded by acidic forms of SMase and by different glycosidases (GCase) yielding ceramide that can be further hydrolyzed by the acid ceramidases (CDase) **(L)**. The sphingosine formed in this reaction can escape the lysosome and can be metabolized to glycerophospholipids after phosphorylation by SK1 and cleavage by S1P lyase (SGPL) or it can be recycled for sphingolipid synthesis in the salvage pathway **(M)**.

Ceramide, synthesized in the endoplasmic reticulum (ER), is the common precursor for the biosynthesis of SM and glycosphingolipids (**Figure 2**). In turn, degradation of SM and glycosphingolipids at the lysosomal level or in another subcellular localization can yield ceramide. Lysosomal ceramide is the key intermediate of complete catabolism of complex sphingolipids. Sphingosine, generated by ceramide hydrolysis, can be phosphorylated from two known sphingosine kinases (SK1 and SK2) to produce sphingosine 1-phosphate (S1P) (**Figure 1**), which is irreversibly cleaved by an S1P lyase, yielding phosphoethanolamine and a fatty aldehyde (**Figure 2**). For decades, ceramide, sphingosine, and S1P have been regarded solely as intermediates in complex sphingolipid metabolism. However, from the end of the 1980s, a number of papers appeared, describing the effects of these molecules on a plethora of cellular targets, implying their involvement in several biological processes. For these relatively simple sphingosine-based molecules, the term “bioactive sphingoids” has been coined.

Stimulus-mediated SM hydrolysis by sphingomyelinases (SMases) (Hannun, 1994) was described as the main source of bioactive ceramide. The production of bioactive ceramide is not necessarily a lysosomal event because SMases are present at the plasma membrane or can be recruited to it from intracellular sites as a consequence of different stimuli (Levade and Jaffrezou, 1999; Goni and Alonso, 2002). Ceramide, acting on specific cellular targets (including several signaling protein kinases and phosphoprotein phosphatases) or determining the reorganization of plasma membrane signaling platforms (Zhang et al., 2009), regulates several cellular events, most notably programmed cell death with important consequences in cancer, including inflammation, bacterial infection, and signaling pathways related to Alzheimer’s disease (AD) and other neurodegenerative and neurological disorders (Van Echten-Deckert and Walter, 2012; Czubowicz et al., 2019). On the other hand, ceramide can be phosphorylated by a specific ceramide kinase yielding ceramide 1-phosphate, another bioactive molecule acting on its own cellular targets (Presa et al., 2016). Ceramide hydrolysis by various ceramidases negatively regulates ceramide levels; however, it fuels the production of S1P by providing sphingosine as a substrate for sphingosine kinases. As already mentioned, S1P derived from the catabolism of ceramide and more complex sphingolipids is an important biologically active mediator involved in diverse signal transduction pathways that regulate many different cell functions, in some cases, with effects opposed to those of ceramide. Considering the high numbers of enzymes, transporters, cellular targets, and receptors involved in the regulation of the cellular levels of bioactive sphingoids, and in their cellular actions, they do represent an intriguing druggable target for many pathologies.

## Sphingosine 1-Phosphate Metabolism and Signaling

As mentioned above, ceramide-derived sphingosine can either be recycled for the resynthesis of sphingolipids or be phosphorylated at C1 with the generation of S1P by two isoforms of sphingosine kinase, sphingosine kinase 1 (SK1), and sphingosine kinase 2 (SK2). S1P can be metabolized through irreversible cleavage by the S1P lyase enzyme (Serra and Saba, 2010) (SGPL1) to a fatty aldehyde (hexadecenal if starting from the most common C18 sphingosine) and phosphoethanolamine; alternatively, S1P can also be dephosphorylated back to sphingosine through a reaction that is catalyzed by lipid phosphatase or S1P-specific phosphatases (Le Stunff et al., 2002; Le Stunff et al., 2007; Pyne et al., 2009; Giussani et al., 2014).

**Figure 3:**
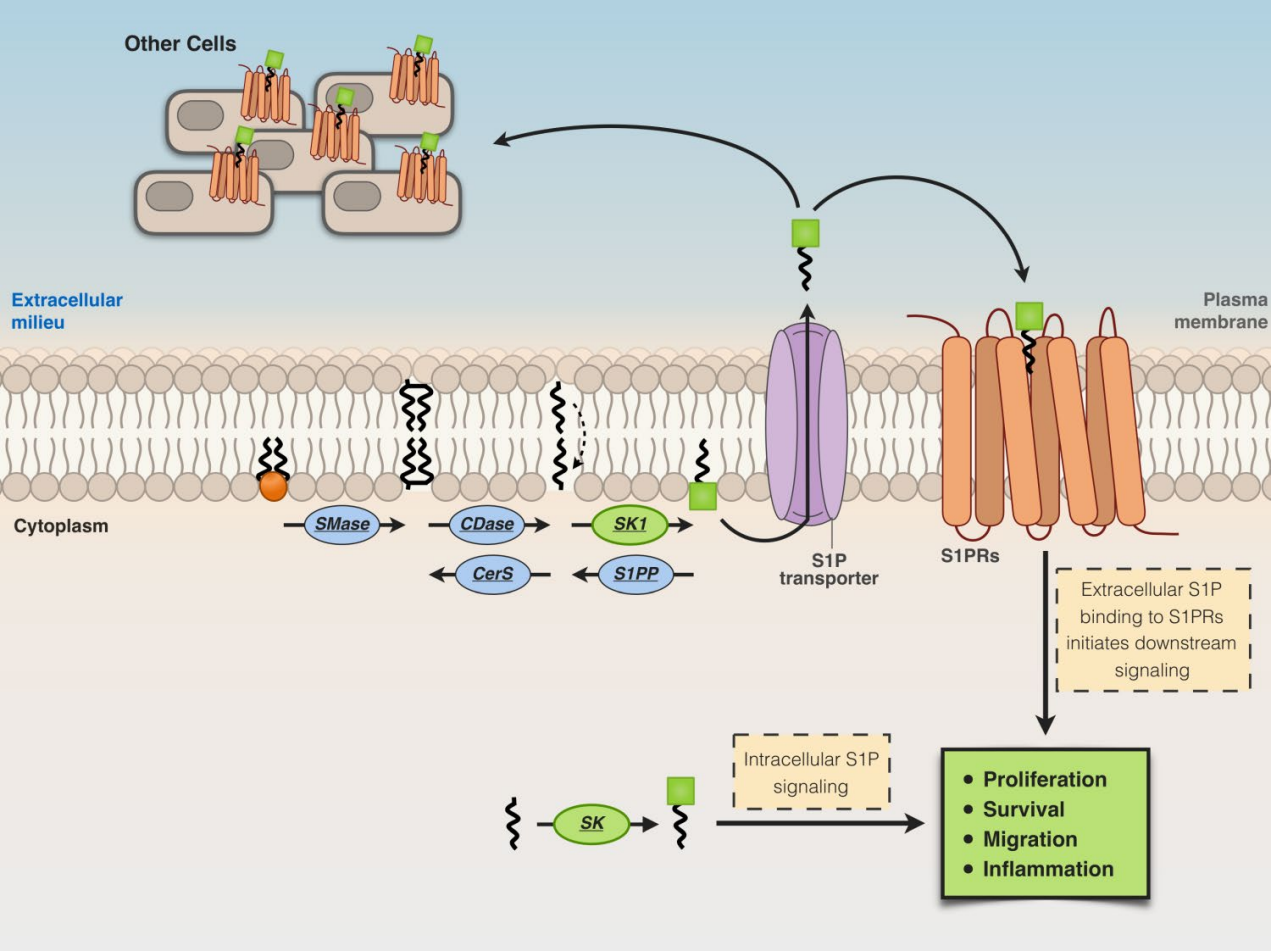
Extracellular and intracellular actions of S1P. Cells release S1P in the extracellular milieu through specific transporters [spinster homolog 2 (Spns2) or ABC transporters]. S1P in the extracellular milieu then binds to S1P receptors (S1P_1_–S1P_5_) located at the plasma membrane, thus inducing biological responses.

S1P is released from cells in the extracellular milieu through specific transporters [spinster homolog 2 (Spns2) or ABC transporters], then from the extracellular milieu, S1P binds to a family of plasma membrane G protein-coupled receptors, the S1P receptors (S1P_1_–S1P_5_) (Giussani et al., 2014; Pyne et al., 2016), triggering different biological responses (**Figure 3**). On the other hand, S1P generated intracellularly by the action of SK2 can engage to various targets, including HDAC-1/2 (Hait et al., 2009), human telomerase reverse transcriptase (hTERT) (Panneer Selvam et al., 2015), and prohibitin 2 (Strub et al., 2011). Thus, the activity of SK2 seems relevant for diverse and crucial biological events, including epigenetic regulation, aging, and mitochondrial respiratory complex assembly (Pyne et al., 2017) (**Figure 3**).

It is now known that S1P metabolism is tightly regulated. In particular, new pieces of evidence indicating specific roles for SK1 and SK2 in different diseases, including cancer, cardiovascular system and central nervous system (CNS) affections, inflammation, and diabetes, have been recently published (Pyne et al., 2016; Cattaneo et al., 2018). In particular, the role of S1P/SK2 pathway in regulating the survival of the dopaminergic neurons has emerged, suggesting that dysregulation of this pathway might be relevant to the clinical development of Parkinson’s disease (PD); in addition, a role for SK1 and SK2 has also been suggested in the onset of AD (Pyne et al., 2016). Moreover, Hagen et al., (2009) demonstrated that there is an additional aspect of S1P generated by SK2 that may exert a neurotoxic effect, inducing apoptosis in lyase-deficient neurons.

Moreover, the involvement of S1P/S1P lyase in cystic fibrosis pathology is supported by the evidence that in a murine cystic fibrosis model, treatment with a specific SGPL1 inhibitor is able to alter S1P metabolism and results in a reduction of the lung inflammatory response to *Pseudomonas aeruginosa* (Veltman et al., 2016).

S1P is a positive regulator of cellular survival, proliferation, and motility in glial cells (Bassi et al., 2006). In particular, it has been shown that S1P is able to mediate calcium signaling in cultured cerebellar astrocytes, whereas it failed to trigger any calcium-mediated response in differentiated cerebellar neurons. This effect could be important for neuron–glia communication in the cerebellum (Giussani et al., 2007).

On this basis, it is crucial to continue research to develop isoform-selective SK inhibitors (**Table 1**).

**Table 1:** Research and clinical use of enzymes of S1P metabolism.

Inhibitor	Target	Indications for diseases	Reference
SKI-II	SK1 and SK2	Cancers	French et al., 2006; Santos and Lynch, 2015
SK1-I	SK1	Cancers	Paugh et al., 2008; Kapitonov et al., 2009
PF543	SK1	Sickle cell disease Cancers	Zhang et al., 2014
ABC294640	SK2	Cancers Lupus nephritis Osteoarthritis Diabetic retinopathy Crohn’s disease Rheumatoid arthritis Ulcerative colitis	Santos and Lynch, 2015 Snider et al., 2013 Fitzpatrick et al., 2011b Maines et al., 2006 Maines et al., 2010 Fitzpatrick et al., 2011a Maines et al., 2008
K145	SK2	Cancers	Liu et al., 2013b
LX-2931	S1P lyase	Cystic fibrosis	Veltman et al., 2016

Literature has recently shown a cytotoxic effect of S1P (somewhat in contrast with the mainstream ceramide/S1P rheostat theory) in neurons (Hagen et al., 2011) and in beta-cells of the pancreas (Hahn et al., 2017). In particular, it has been demonstrated that S1P induced i) apoptosis in hippocampal neurons (Moore et al., 1999) as well as ii) accumulation of S1P-induced apoptosis in neurons lacking S1P lyase (Hagen et al., 2009). Moreover, Hagen et al. (2011) have shown that cerebellar neurons with abundant S1P lyase expression are the first to degenerate in S1P lyase-deficient mice. These findings give light to an important role of S1P lyase, which is ubiquitously expressed, in the regulation of physiological and pathophysiological aspects, as suggested also in the review by Choi and Saba (2019).

## Sphingosine 1-Phosphate in Neuroinflammation and Neurodegeneration

Most acute or chronic neurodegenerative diseases are associated with changes in the total levels and/or in the composition of sphingolipids in different areas and cell populations in the nervous system. However, several questions remain without any answer on these sphingolipid alterations. Some sphingolipids are bioactive sphingolipids that are involved in the regulation of cell fate. Among them, S1P mediates crucial signaling pathways relevant to neurodegeneration (Hagen et al., 2011; Assi et al., 2013; Blaho and Hla, 2014; Proia and Hla, 2015; Pyne et al., 2018; Wang and Bieberich, 2018). In the brain, S1P regulates several fundamental processes, such as proliferation, survival, differentiation, and migration of all the different cell populations, including neurons, astrocytes, microglia, and oligodendrocytes, as well as infiltration of peripheral immune cells in the CNS during neuroinflammation (Kleger et al., 2007; Riccitelli et al., 2013; Smith et al., 2013; Marfia et al., 2014; Chiricozzi et al., 2018; Grassi et al., 2019). S1P can act both as an extracellular and as an intracellular mediator (Hait et al., 2009; Alvarez et al., 2010; Blaho and Hla, 2014; Proia and Hla, 2015; Park et al., 2016). It regulates different signal transduction pathways in a cell type- and context-specific manner. When acting as an extracellular mediator, S1P effects are dependent on the type and expression levels of the different S1P receptor(s). Different S1P receptors are coupled to different G-proteins, thus regulating specific signaling pathways, including those mediated by MAPK, PI3K/Akt, and phospholipase C (Spiegel et al., 1998; Huang et al., 2011; Spiegel and Milstien, 2011; Kunkel et al., 2013; Blaho and Hla, 2014; Proia and Hla, 2015). On the other hand, different S1P intracellular targets have been recently discovered in several cell types, including neurons. It has been demonstrated in different cell types that S1P regulates the function of some proteins in different cellular compartments, such as the ER and nuclei; for example, HDACs, TRAF2, Hsp90, and HRP94 (Hait et al., 2009; Alvarez et al., 2010; Blaho and Hla, 2014; Proia and Hla, 2015; Park et al., 2016). In particular, three different nuclear transcription factors seem to be regulated by S1P-dependent pathways: 1) FOXO3a, which in PC12 cells is inhibited by the PI3K/Akt pathway (Safarian et al., 2015); 2) AP-1, regulated by the Jnk/p38/ERK pathway (Hsu et al., 2015; Jazvinscak Jembrek et al., 2015) and, in turn, controlling the mutual coregulation between sphingolipid-related genes (O’Neill et al., 2011; Huang et al., 2014; Wegner et al., 2014); 3) NF-κB, which is regulated *via* the tumor necrosis factor (TNF) receptor-associated factor TRAF2. In fact, TRAF2 can directly interact with SK1 and, on the other hand, it can be regulated by S1P as a receptor cofactor (Xia et al., 2002; Alvarez et al., 2010). In addition, histone deacetylases (HDAC1 and HDAC2), which can negatively regulate NF-κB *via* its deacetylation (Dai et al., 2005), are inhibited through S1P binding (Hait et al., 2009). The final effect of NF-κB influence on cell fate may vary, depending on the particular signaling context and by the underlying immune activation, among many other factors. Finally, S1P, by acting on its receptors and affecting the downstream PI3K/Akt, can inhibit GSK-3β (the crucial tau kinase) (Wang et al., 2015) and the proapoptotic protein Bad (Czubowicz et al., 2019).

It is now known that S1P has physiological functions in the CNS; several papers demonstrate that S1P plays an essential role in brain development (Mizugishi et al., 2005; Blaho and Hla, 2014; Proia and Hla, 2015). Mizugishi et al. (2005) demonstrated that SK1 is highly expressed in mouse brain during normal development. Moreover, S1P depletion in SK1/SK2-double knockout mice (Mizugishi et al., 2005) caused severe defects in neural cell survival and in neurogenesis, leading to increased neural cell apoptosis and impaired neural tube closure, ultimately leading to an embryonic lethal phenotype. Remarkably, S1P_1_-null mice show a very similar neural phenotype, suggesting a pivotal role of S1P signaling in neurogenesis and brain development (Mizugishi et al., 2005; Blaho and Hla, 2014; Proia and Hla, 2015).

Abundant literature suggests important roles of S1P also for neuronal survival and for the maintenance of neuronal functions in adult brains. Different papers demonstrate that in hippocampal neurons, S1P is important for the stability of the presynaptic structure, for synaptic strength, and for the availability of synaptic vesicles (Brailoiu et al., 2002; Camoletto et al., 2009; Darios et al., 2009; Kanno et al., 2010; Riganti et al., 2016). On the other hand, under certain circumstances, S1P might become harmful. In fact, Mitroi et al. (2016) demonstrated that a certain S1P threshold concentration causes an increase in basal calcium in neurons that impairs presynaptic architecture most probably *via* a UPS-mediated mechanism.

Moruno Manchon et al. (2015, 2016) demonstrated that S1P promotes prosurvival neuronal autophagy; in particular, they have shown that SK1 enhances autophagic activity, whereas SGPL1 reduces this activity. Autophagy plays a crucial role for neuron survival; in fact, this process allows neurons to get rid of damaged and aggregated proteins and organelles during different conditions, such as aging and diseases, in particular, to counteract cell death induced by ceramide or other pathogens (Moruno Manchon et al., 2015; Moruno Manchon et al., 2016; Moruno-Manchon et al., 2018).

However, the role of S1P metabolism in brain autophagy is very complex. In fact, in contrast to these results, Mitroi et al. (2017) demonstrated that the deficiency of SGPL exerted a block on the autophagic flux. The authors demonstrated that depletion of SGPL, increasing S1P levels while decreasing ethanolamine phosphate and consequently phosphatidylethanolamine, blocks autophagy at the initial stages (Mitroi et al., 2017). These findings suggest that in neurons, the increase of S1P and the simultaneous decrease of phosphatidylethanolamine caused by the modulation of SGLP both affect autophagy even if in an antagonistic way (Mitroi et al., 2017).

Altogether, the role of S1P as a bioactive lipid in the nervous system appears to be dual (Van Echten-Deckert and Alam, 2018). On one hand, as described above, it is essential for proper brain development; on the other hand, its detrimental effects and cytotoxic effects on some particular neuronal populations have been documented (Van Echten-Deckert et al., 2014).

The regulation of S1P concentration in the different cerebral areas suggests a specific function of S1P in different brain regions.

Recently, thanks to the approval by the Food and Drug Administration (FDA) of the sphingosine analog and S1P_1_ functional antagonist fingolimod {FTY720, Gilenya, 2-amino-2[2-(4-octylphenyl)ethyl]-1,3-propanediol}, the functions of S1P in neuroinflammation processes regarding neurodegeneration attracted the attention of the scientific community. Microglia activation is emerging as one of the crucial factors contributing to the onset of neuroinflammation associated with different neurodegenerative diseases.

Nayak et al. (2010) demonstrated that in activated microglia, SK1 expression is upregulated. In turn, SK1 affects the production of proinflammatory cytokines and nitric oxide in lipopolysaccharide (LPS)-treated microglia (Nayak et al., 2010). In other cell types, treatment with LPS was able to activate the SK1/S1P signaling pathway by inducing the translocation of SK1 to the plasma membrane, leading to increased production of S1P (Hammad et al., 2008; Fernandez-Pisonero et al., 2012). The administration of exogenous S1P to activated microglia increased the inflammatory response, inducing the production of proinflammatory cytokines and neurotoxins (Assi et al., 2013; Lv et al., 2016). Altogether, data in the literature suggest that the S1P/SK1 pathway, acting as an autocrine or paracrine factor, is involved in the inflammatory response of activated microglia, regulating the release of proinflammatory factors by microglia.

On the other hand, it has been shown that under proinflammatory conditions, SK1 and S1P_3_ in astrocytes are functionally upregulated (Fischer et al., 2011). Moreover, treatment of astrocytes with IL-1 induces the expression of SK1 and, in turn, exposure to exogenous S1P induces astrogliosis (Sorensen et al., 2003; Paugh et al., 2009). In a mouse model of multiple sclerosis (MS), it has been shown that particular clusters of astrocytes are activated, and astrocyte activation progressively expand along white matter tracts. Moreover, the loss of astrocytic S1P_1_ limited the extent of astrocyte activation (Choi et al., 2011; Dusaban et al., 2017). The role of S1P_1_ in astrocytes seems to be essential not only for astrocyte’s own functions but also for the interplay between astrocytes and other cell types. Indeed, modulation of S1P_1_ by fingolimod in astrocytes effectively suppressed different neurodegeneration-inducing pathways mediated by astrocytes, but also by microglia, and by CNS-infiltrating activated leukocytes (Karunakaran and Van Echten-Deckert, 2017; Rothhammer et al., 2017; Wang and Bieberich, 2018).

Considering the role of S1P in the development and physiological homeostasis of the nervous system and its emerging importance in neuroinflammation, it is not surprising that dysregulation of S1P metabolism and S1P-mediated signaling is emerging as a common trait and an important causative factor in various neurodegenerative diseases, including MS, AD, PD, and Huntington disease (HD). The knowledge about the role of S1P in these affections is briefly outlined in the following sections of this review.

## Sphingosine 1-Phosphate and Multiple Sclerosis

MS is a chronic inflammatory disease of the CNS, in which the inflammatory process is associated with a destruction of myelin, leading to the appearance of large focal lesions of demyelination. Axonal damage and loss as consequences of the inflammatory demyelination also occur, even if at variable extents. Active remyelination processes can at least in part repair myelin lesions, whereas axonal loss is permanent and irreversible.

MS is primarily considered an autoimmune neurodegenerative disease, that is, a disease caused by an adaptive immune response to self-antigens. In MS, activated myelin-reactive T cells [in particular, T helper 1 cells (Th1)] are recruited from the periphery to the CNS, leading to the activation of microglia and to the recruitment of circulating macrophages. Consistently with this, fingolimod has proven to be an effective disease-modifying drug for the treatment of relapsing–remitting MS (RR-MS).

**Figure 4:**
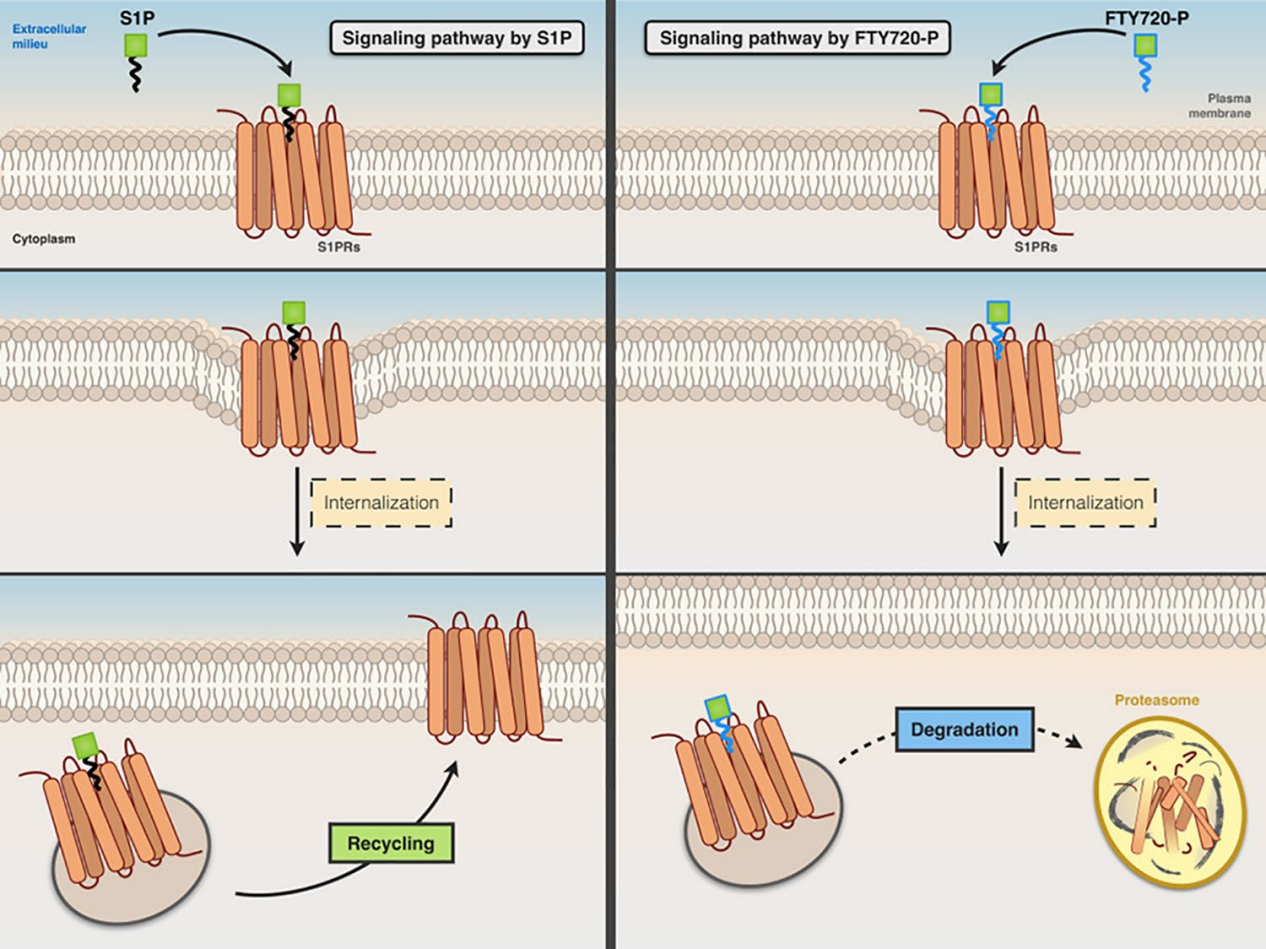
Comparative signaling pathways of S1P and FTY720-P. Both S1P (left panel) and FTY720-P (right panel) stimulate the internalization of S1P receptor. The receptor that binds S1P is recycled back to the cell surface, whereas FTY720-P causes irreversible internalization and degradation of bound S1P receptor.

Fingolimod, a structural analog of sphingosine, is converted *in vivo* to fingolimod-P, a structural analog of S1P, which acts as a nonselective agonist for S1P_1_, S1P_3_, S1P_4_, and S1P_5_ receptors (lacking activity on S1P_2_). It acts as a functional antagonist of S1P receptors, causing the irreversible internalization and degradation of bound S1P receptors (thus preventing their recycling back to the cell surface) (**Figure 4**).

Fingolimod is effective on MS by preventing the recruitment of T cells, expressing S1P_1_ and S1P_3_ receptors, with their consequent redistribution to secondary lymphoid organs, and preventing invasion of auto-aggressive T cells to the CNS. However, the heterogeneity of human MS and the comparison between the human disease and the different animal models suggest that additional factors other than Th1-mediated autoimmunity are relevant to lead from primary demyelination to a chronic inflammatory lesion. It is worth to note that primates, differently from mice, do not develop demyelination because of pure T cell-mediated inflammation. In fact, fingolimod effectively decreased astrocyte and microglia activation in a non-T cell animal model of demyelination, the cuprizone mouse model (Kim et al., 2011).

A significant contribution to the progression of the disease is likely given by circulating demyelinating antibodies against myelin surface components, most notably the anti-myelin oligodendrocyte glycoprotein antibodies, occurring in a significant proportion of MS patients (Linington et al., 1988; Ramanathan et al., 2016; Spadaro et al., 2018). In addition, cell populations resident in the lesion niche, most notably astrocytes and microglia, might critically affect both oligodendrocyte injury and axonal degeneration (Mayo et al., 2012). Indeed, changes in the expression of 13 different growth factors, known to regulate the development and maintenance of oligodendrocytes, were highlighted during demyelination and remyelination in the murine cuprizone model of toxic demyelination (Gudi et al., 2011). In particular, in lesion areas, IGF-1 and CNTF were elevated in astrocytes, whereas GDNF, IGF-1, and FGF were detected at high levels in microglia.

In MS, oligodendrocyte loss and myelin damage can be observed in early lesions, even in the absence of infiltrates of immune cells from the periphery. On the other hand, early activation and proliferation of microglia and astrocytes are consistently present in MS lesions. Several pieces of evidence suggest that S1P signaling in these cell populations might be relevant to the progression of the disease, opening up new perspectives for drugs acting on the S1P axis in the therapy of MS (reviewed in Groves et al., 2013; Farez and Correale, 2016).

Altered sphingolipid metabolism and altered sphingolipid-dependent signaling in reactive astrocytes might contribute to oligodendrocyte damage in MS (Kim et al., 2012). Ceramide accumulated in reactive astrocytes in active lesions of MS and in a non-T cell animal model of demyelination (the cuprizone mouse model). Ceramide accumulation was consequent to the upregulation of serine palmitoyltransferase in reactive astrocytes. In culture, ceramide acted synergistically with TNF, inducing apoptosis of oligodendrocytes (an astrocyte-dependent event). Concomitantly, sphingosine was accumulated whereas S1P levels were decreased. These alterations in sphingolipid metabolism were restored upon active remyelination.

It has been shown that sphingosine kinase 1 and S1P_3_ are upregulated in reactive astrocytes present at the lesion site or in cultured rat astrocytes treated with LPS. S1P induced secretion of CXCL1 in astrocytes, and secretion was increased in astrocytes pretreated with LPS. Thus, ceramide/S1P pathway in astrocytes is relevant for astrocyte activation, and in MS, it could be detrimental, enhancing astrogliosis, or beneficial, through increased remyelination sustained by CXCL1 (Fischer et al., 2011).

As mentioned above, the main therapeutic effect of fingolimod in MS seems to be related to its ability to prevent migration of auto-aggressive lymphocytes to the CNS. However, because S1P receptors are widely expressed in the CNS, fingolimod easily crosses the blood–brain barrier (BBB) and the effect on MS is at least in part independent of the effect on the migration of immune cells from the periphery (Meno-Tetang et al., 2006; Foster et al., 2007; Chun and Hartung, 2010).

In fact, emerging evidence indicates that the efficacy of fingolimod in MS is at least in part caused by its direct effects on the CNS. S1P signaling effects relevant for MS likely involve different neural cell types (astrocytes, oligodendrocytes, neurons, microglia, and dendritic cells); however, very strong evidence indicates a primary role of astrocytes in the direct effects of fingolimod on the CNS. Astrocytes express S1P receptors, mainly S1P_1_ and S1P_3_. S1P_1_ and S1P_3_ are upregulated in reactive astrocytes present in demyelinating and chronic MS lesions. S1P modifies astrocyte morphology and increases the expression of GFAP, marker of astrogliosis. Fingolimod stimulates migration of cultured astrocytes, whereas it acts *in vivo* as a functional antagonist of astrocyte S1P_1_.

In experimental autoimmune encephalomyelitis (EAE), an experimental paradigm of Th1-mediated demyelinating disease, fingolimod is highly effective; however, its effects are abolished in animals selectively lacking the expression of S1P_1_ in astrocytes (even if the receptor expression and function in immune cells are preserved) (Choi et al., 2011). As an endpoint, fingolimod appears to be able to promote remyelination by acting on oligodendrocytes, microglia, or astrocytes. In addition, fingolimod was effective in increasing remyelination in a rat CNS reaggregate spheroid cell culture model (a CNS environment devoid of immune system effects) upon lysoPC-induced transient demyelination. Increased remyelination was associated with partial inhibition of microglia activation (Jackson et al., 2011). In summary, downmodulation of S1P_1_ receptors in astrocytes upon fingolimod treatment causes reduced astrocyte activation and improves the communication of astrocytes with other CNS cells, resulting in reduced demyelination or improved remyelination.

S1P signaling as a pharmacological target in MS is relevant not only in astrocytes but also in microglia. As mentioned above, microglia can contribute to the release of factors positively affecting remyelination. On the other hand, fingolimod in cultured microglia was able to suppress *via* binding of S1P_1_ the production of relevant proinflammatory cytokines, such as TNFα, IL-1β, and IL-6 (Choi et al., 2011).

A further layer of complexity in the possibility to affect the S1P/S1P receptor axis in MS is revealed by studies suggesting that fingolimod might be effective in modifying some disease parameters by acting as an agonist, rather than a functional antagonist, of given S1P receptors. In addition to motor deficits, over 50% of MS patients suffer from neuropathic pain, a debilitating symptom that dramatically affects the patients’ quality of life. In EAE mice, painlike behavior appears early on and well before the motor symptoms develop. In this model, fingolimod was able to reduce hyperalgesia by acting as an S1P_1_ agonist at the central level, inhibiting spinal nociceptive processing (Doolen et al., 2018). This would suggest that novel site- or receptor-specific drugs could be effective in ameliorating the central neuropathic pain.

Finally, despite the prevailing view of T cell-mediated inflammation as the main culprit in the pathogenesis of MS lesions, some data suggest that the inflammatory response and more in general the contribution of the immune system might have a protective role or a role in the repair of myelin lesions. For example, it has been shown that inflammatory cells within MS lesions might release neurotrophic factors (Kerschensteiner et al., 1999) or factors able to stimulate the physiological, *per se* insufficient, mechanism or remyelination (Diemel et al., 1998). On the other hand, some naturally occurring autoantibodies, a subset of monoclonal antibodies that is a part of the normal immunoglobulin repertoire, were able to stimulate remyelination in CNS demyelinating diseases (Rodriguez et al., 2009). rHIgM22, the recombinant form of a naturally occurring antibody isolated from a patient with Waldenström macroglobulinemia, was able to bind selectively to myelin *in vitro* and to the surface of cultured oligodendrocytes. It was shown to enter the CNS and to accumulate at the lesion sites in MS (Pirko et al., 2004) and to promote remyelination in Theiler’s murine encephalomyelitis virus (Warrington et al., 2000)-, lysolecithin (Bieber et al., 2002)-, and cuprizone-induced (Mullin et al., 2017) demyelination models of MS. A 16-site Phase 1 clinical trial in MS patients completed in 2015 (NCT0183867) showed no dose-limiting toxicities, no serious treatment-emergent adverse events, and the presence of the antibody in the CFS in all patients (Eisen et al., 2017). Although some studies suggest that the rHIgM22 remyelination-promoting effect is exerted directly on myelin-producing cells (Watzlawik et al., 2010; Watzlawik et al., 2013), others suggest that it acts on other cell types present in the lesion niche, including astrocytes (Paz Soldan et al., 2003) and microglia (Zorina et al., 2018). We recently showed that rHIgM22 stimulated the proliferation of astrocytes in mixed glial cells because of the increased production and release of S1P, suggesting that S1P signaling might be significant in the interplay of different cell types involved in the complex series of events eventually leading to myelin repair (Grassi et al., 2019).

## Sphingosine 1-Phosphate and Alzheimer’s Disease

AD is hallmarked by the accumulation of intraneuronal aggregates of hyperphosphorylated tau protein and deposition of extracellular oligomer amyloid beta peptide (predominantly Aβ42 and Aβ40). Evidence strongly suggests a critical role of Aβ, which derives from the proteolytic processing of the amyloid precursor protein (APP), in the initiation of AD (Kozlov et al., 2017).

The possible role of S1P in AD is currently quite controversial. There is evidence suggesting a direct role of S1P in the initiation and progression of AD. S1P was found to directly interact and stimulate the proteolytic activity of the β-secretase BACE1, the rate-limiting enzyme for Aβ production. Moreover, overexpression of SGPL1 and inhibition or knockdown of SKs reduced BACE1 activity, *in vitro* and *in vivo*, with consequent decreased Aβ secretion, suggesting a correlation between levels of S1P and those of Aβ (Hagen et al., 2011; Takasugi et al., 2011). A study of SGPL1-deficient mice also revealed that S1P elevated levels to exert a neurotoxic effect mediated by a calcium/calpain/CDK5 mechanism (Hagen et al., 2011). Moreover, SGPL1 deficiency was also associated with hyperphosphorylation of tau, accumulation of APP, and impaired lysosomal activity (Hagen et al., 2011; Karaca et al., 2014). AD brains also showed an increased SK2 activity (Takasugi et al., 2011), and ERK and Fyn kinase, both known modulators of this enzyme, have been implicated in Aβ-mediated neurotoxicity (Olivera et al., 2006; Hait et al., 2007; Crews and Masliah, 2010), which could suggest that SK2 upregulation might be mediated by aberrant phosphorylation by Fyn and/or ERK. On the other hand, consistently with the prosurvival and antiapoptotic effects of S1P, the S1P/sphingosine ratio is decreased in postmortem AD brains and hippocampus, and the decrease is associated with the reduction of SK1 activity. Increased levels of SGPL1 and S1P phosphatase have also been reported in these brains (Katsel et al., 2007; He et al., 2010; Ceccom et al., 2014; Couttas et al., 2014), indicating a protective role of S1P in AD. SKs have also been shown to exert a protective influence in Aβ toxicity. Their overexpression reduced the toxic effects of Aβ (Gomez-Brouchet et al., 2007) whereas SK1 silencing in APP/PS1 mice led to an increased secretion of Aβ and, consequently, an increased Aβ-induced cell death (Zhang et al., 2013). Suppression of SK1 by miRNA 125b, which is abundant in AD, correlated with an increased Aβ production *in vitro* (Jin et al., 2018).

The exact mechanism through which S1P could exert its protective role is still unclear; however, a few studies (Gomez-Munoz et al., 2003; Malaplate-Armand et al., 2006; He et al., 2010) suggest that S1P might act by inhibiting the activation of acid sphingomyelinase, whose activity has been found to be increased in AD brains (Gomez-Munoz et al., 2003).

SK2 also has a controversial role in AD. Some authors, as previously mentioned, showed an increase in SK2 activity in the frontal cortex of AD brains (Takasugi et al., 2011), whereas others reported a decreased activity in temporal cortex and hippocampus (Couttas et al., 2014). These results, however, may simply reflect the complexity of SK regulation and function, and subcellular localization may play a role in SK2 expression in AD. Interestingly it has been observed that in AD brains, the equilibrium between the nuclear and the cytoplasmic SK2 was altered. It has been reported that SK2 in these brains preferentially localized in the nucleus, whereas the cytoplasmic expression of the enzyme inversely correlated to Aβ deposits (Dominguez et al., 2018).

Fingolimod, the immunomodulatory analogue of S1P, has been tested in AD models. Fingolimod was able to reduce the S1P Aβ-induced neuronal damage in rat hippocampus and improve the consequent cognitive impairment (Asle-Rousta et al., 2013a, Asle-Rousta et al., 2013b). In a mouse model of AD, treatment with fingolimod lad to a reduction of soluble and insoluble Aβ and to decreased Aβ plaque density (Aytan et al., 2016). Furthermore, both fingolimod and KRP203, another S1P analogue, decrease Aβ generation in neuronal cells (Takasugi et al., 2013). The effect of fingolimod was also tested in transgenic mice overexpressing APP and presenilin 1 (APP/PS1 mice). In this model, Aβ accumulation and loss of neuronal function are coupled with augmented BBB permeability and with the activation of astrocytes and microglia (Kelly et al., 2013; Mcmanus et al., 2014; Minogue et al., 2014). A reduction in both accumulation of Aβ and astroglial activation was observed after treatment with fingolimod. Moreover, the treatment also increased the phagocytic activity of astrocytes, suggesting that the decrease in Aβ accumulation in the treated mice could be a consequence of this enhanced phagocytic function (Mcmanus et al., 2017).

Fingolimod’s exact mechanism in AD however remains to be elucidated; however, the protective effect of fingolimod in neurons was associated with an altered expression of MAPKs and some inflammatory markers (Hemmati et al., 2013).

## Sphingosine 1-Phosphate and Parkinson’s Disease

PD is one of the most common neurodegenerative disorders, second only to AD, and is clinically defined by the degeneration or death of dopaminergic neurons in the *substantia nigra* accompanied by the formation of aggregates of alpha-synuclein and ubiquitin, called Lewy bodies (Beitz, 2014).

In neuronal cells (SH-SY5Y) treated with 1-methyl-4-phenylpyridinium (MPP+), an active metabolite of MPTP (1-methyl-4-phenyl-1,2,3,6-tetrahydropyridine), there is a reduction in SK1 gene expression and protein levels, with a concomitant increase of SGPL1 that leads to enhanced reactive oxygen species (ROS) generation (Pyszko and Strosznajder, 2014a; Pyszko and Strosznajder, 2014b). Moreover, pharmacological inhibition of SK1 was shown to suppress prosurvival PI3K/Akt phosphorylation, activation and upregulation of proapoptotic proteins, cytochrome c release from mitochondria, and caspase-dependent apoptosis in these cells (Pyszko and Strosznajder, 2014a; Pyszko and Strosznajder, 2014b). In the same experimental model, inhibition of SK1 and SK2 also led to an increased secretion of alpha-synuclein, a protein able to negatively regulate S1P_1_ signalling (Pyszko and Strosznajder, 2014a; Pyszko and Strosznajder, 2014b; Badawy et al., 2018). Interestingly, treatment with pramipexole, a D2/D3 receptor agonist commonly used in PD therapy, partially reversed SK1 inhibition in the MPP+ model (Motyl et al., 2018).

In MPP+-treated neuronal cells, S1P administration showed neuroprotective effects. The treatment with exogenous S1P in fact significantly increased cell viability mainly through activation of S1P_1_ receptor-mediated signalling, reduced the mRNA level of proapoptotic proteins such as Bax and Hrk, and decreased cytochrome c levels in a mitochondrial fraction, leading to caspase-dependent poly(ADP-ribose) polymerase-1 proteolysis (Pyszko and Strosznajder, 2014a; Pyszko and Strosznajder, 2014b). Interestingly, *S1P* *_1_* was recently proposed as a candidate gene for a newly identified PD susceptibility locus (Hill-Burns et al., 2014), supporting the potential role for S1P_1_ signalling in regulating midbrain dopaminergic neuron survival and/or function. Phospholipid phosphatase 3, an integral membrane glycoprotein involved in the modulation of S1P metabolism and signalling in the brain (Lopez-Juarez et al., 2011), has also been implicated in PD, and its inactivation in CNS progenitor cells caused a severe downregulation of S1P_1_ in the adult ventral midbrain and in the cerebellum (Lopez-Juarez et al., 2011; Gomez-Lopez et al., 2016).

A marked decrease of SK2 levels has been reported in the *substantia nigra* of the 1-methyl-4-phenyl-1,2,3,6-tetrahydropyridine (MPTP)-treated C57BL/6 mice, a PD mouse model (Sivasubramanian et al., 2015). Moreover, SK2 inhibition in MN6D dopaminergic neurons decreased the expression of genes for the regulation of mitochondrial function and led to marked ATP depletion and reduction of superoxide dismutase 2 levels. An increase in the ROS levels was also observed (Sivasubramanian et al., 2015).

Administration of fingolimod protected against neurodegeneration and behavioral effects in mouse PD models induced by MPTP, 6-hydroxydopamine, or rotenone through S1P_1_ signalling and probably Akt (Ren et al., 2017; Zhao et al., 2017; Motyl et al., 2018); however, the S1P analogue was not effective in a PD model induced by subacute administration of MPTP (Komnig et al., 2018). Cellular studies applying newly modified versions of fingolimod referred to as C2 moiety (FTY720 C2 or FTY720-Mitoxy) have shown that these compounds increase BDNF levels, activate protein phosphatase 2A, whose activity is impaired in PD, and protect MN9D cells against TNFα-induced cell death (Vargas-Medrano et al., 2014). Long-term oral administration of fingolimod reduced alpha-synuclein aggregation and increased BDNF levels in transgenic mice overexpressing mutant human alpha-synuclein (Vidal-Martinez et al., 2016). There are also several reports indicating a fingolimod neuroprotective effect in PD models induced by toxins. For example, in C57BL/6 mice subjected to acute MPTP intoxication, there was a reduced expression and activity of SK1. This study also showed that fingolimod exerts neuroprotective effects comparable to pramipexole, a dopamine D2/D3 receptor agonist (Motyl et al., 2018).

## Sphingosine 1-Phosphate and Huntington’s Disease

HD is an inherited neurodegenerative brain disease characterized by the progressive degeneration of the striatum and cortex, with consequent motor, cognitive, and behavioral symptoms. It is caused by a dominant mutation, which leads to the expansion of a CAG trinucleotide repeat in the gene encoding for the huntingtin protein. The consequence of the mutation is the expression of a protein with a polyglutamine stretch in the *N*-terminal region much longer than in the wild type (Jimenez-Sanchez et al., 2017). The exact biological function of huntingtin is poorly understood, but it is highly expressed in brain where it seems to be relevant for neuronal development and metabolism.

Recent studies indicate that the expression of S1P-metabolizing enzymes is altered in several HD models, including animal models, human brain tissues, and cultured cells (Di Pardo et al., 2017; Pirhaji et al., 2017). Upregulation of SGPL1 and reduced expression of SK1 are detectable in human postmortem brains and in striatal tissues of two of the most commonly used HD animal models (R6/2 and YAC128 mice), even at an early stage of the disease (Di Pardo et al., 2017; Pirhaji et al., 2017). Moreover, evidence indicates that miRNA 125b expression, which is downregulated in R6/2 mice, negatively regulates gene expression of SGPL1 (Ghose et al., 2011; Yang et al., 2016). Furthermore, it has been demonstrated that cytoplasmic SK1 is involved in the regulation of huntingtin degradation (Moruno Manchon et al., 2015). R6/2 mice also exhibit increased SK2 and reduced S1P levels (Di Pardo et al., 2017; Pirhaji et al., 2017). This reduction of S1P levels could be a potential therapeutic target. In fact, fingolimod was able to improve neuronal activity and motor function, reduce brain atrophy, and increase R6/2 animal survival (Di Pardo et al., 2014). Moreover, both fingolimod and the pharmacological activator K6PC-6 significantly reduced apoptosis in an HD cellular model and increased the activation of Akt and Erk signalling pathways (Di Pardo et al., 2017), whose regulation is known to be defective in HD (Bowles and Jones, 2014). In R6/2 mice, fingolimod also enhanced phosphorylation of huntingtin at the serine-13/16 residues. This might contribute to the protective effect of fingolimod because this posttranslational modification was associated with reduced toxicity of mutated huntingtin (Atwal et al., 2011; Di Pardo et al., 2014). Moreover, reduced synthesis of GM1 gangliosides was found in HD patients and animal models. Lower GM1 levels were associated with higher sensitivity to neuronal death, and artificial reduction of GM1 synthesis increases the apoptotic rate in normal striatal neurons. It has been shown that GM1 administration to HD mice was able to improve motor symptoms (Di Pardo et al., 2012). Remarkably, the molecular mechanism of this effect seems to be mediated by the GM1-induced phosphorylation of huntingtin. On the other hand, fingolimod was able to restore normal levels of GM1 in HD mice (Maglione et al., 2010; Di Pardo et al., 2014). This is an intriguing finding, suggesting that fingolimod’s effects on sphingolipid metabolism might be much more complex than argued on the basis of its efficacy as a functional antagonist of S1P receptors.

Fingolimod also improved synaptic plasticity and memory in the R6/1 mouse model of HD by regulating BDNF signalling and astroglial reactivity. Fingolimod administration prevented overactivation of NF-κB signalling in R6/1 hippocampus, leading to a decrease in TNFα and induced nitric oxide synthase (iNOS) levels. This reduction correlates with the normalization of p75NTR expression in the hippocampus, consequently preventing p75NTR/TrkB imbalance, a critical mechanism for memory and synaptic function in HD (Brito et al., 2014; Miguez et al., 2015). Moreover, fingolimod increased cAMP levels and promoted phosphorylation of CREB and RhoA in the hippocampus of R6/1 mice, supporting its role in the enhancement of synaptic plasticity (Miguez et al., 2015). Interestingly, these results also provide a possible explanation for fingolimod-induced cognitive benefits in AD because both TNFα and p75NTR are overexpressed in AD patients, and p75NTR downregulation is able to prevent cognitive and neurite dysfunction in an AD mouse model (Hu et al., 2002; Alvarez et al., 2007; Asle-Rousta et al., 2013a; Knowles et al., 2013; Fukumoto et al., 2014).

Modulation of SK2 and SGPL1 may also exert protective effects in HD. Administration of two SK2 selective inhibitors (K145 and EMD56773) markedly reduced apoptosis (Di Pardo et al., 2017). On the other hand, inhibition of SGPL1 (using 2-acetyl-5-tetrahydroxybutyl imidazole or 4-deoxypryridoxine) also reduced cell death. This effect was associated with the inhibition of HDAC activity, which in turn resulted in increased levels of histone H3 acetylation. Because histone H3 deacetylation was previously reported to be reduced in several HD models, this could explain the protective effect of SGPL1 inhibition in this disease (Buckley et al., 2010; Di Pardo et al., 2017; Pirhaji et al., 2017).

SK2 has also been implicated in HDAC regulation. In fact, it has been reported that increasing levels of S1P in the nucleus by overexpressing SK2 lead to histone deacetylases 1 and 2 inhibition. S1P directly binds to these deacetylases while SK2 forms a complex with them, which inhibits their activity (Hait et al., 2009; Hait et al., 2014). Whereas SK2 might exert a neuroprotective role through HDAC inhibition, it has been observed that its overexpression is neurotoxic for cultured striatal and cortical neurons in a dose-dependent manner. In these neuronal models and in the BACHD mouse model of HD, SK2 is hyperphosphorylated and promotes the formation of double-strand breaks. Interestingly, a small molecule inhibitor of SK2, ABC294640, was able to mitigate DNA damage and neurotoxicity and protect against degeneration on two mouse models of HD (Moruno-Manchon et al., 2017).

Stimulation of S1P5 in the R6/2 models using the selective agonist A-971432 was also considered as a potential therapeutic approach for HD. Chronic administration of the agonist slowed down the progression of the disease and prolonged the life span of R6/2 mice. These effects were associated to the activation of the prosurvival pathways (AKT, BDNF, ERK) and with a reduction of mutant huntingtin aggregation. Moreover, A-971432 also protected BBB homeostasis in these mice, and when administered early in the disease, it completely protected them from the classic progressive motor deficit and preserved BBB integrity (Di Pardo et al., 2018).

S1P5 is known to be involved in the regulation of BBB tight junctions, and the effect of its agonist A-971432 was associated with an increased expression of the tight junction proteins occludin and claudin-5 (Feldman et al., 2005; Piontek et al., 2008; Van Doorn et al., 2012).

## Fingolimod and Beyond

The immunomodulatory drug fingolimod (FTY720) is the first drug approved for oral treatment of relapsing-remitting MS (RR-MS) (Kappos et al., 2006; Brinkmann et al., 2010) because of its impressive efficacy and good tolerability. Fingolimod is phosphorylated to form fingolimod-P, very similar to S1P.

Pharmacologically, fingolimod acts as a nonselective agonist of S1P receptors, with the exception of S1P_2_, and as a selective functional antagonist of the S1P_1_ subtype, the main S1P receptor expressed in different tissues, including brain and immune system cells (Blaho and Hla, 2014), inducing receptor downregulation (Pyne et al., 2016). Fingolimod-P initially binds to and activates S1P_1_. After being engaged by fingolimod-P, S1P_1_ is subsequently internalized and degraded (Huwiler and Zangemeister-Wittke, 2018).

Although effective in ameliorating the symptoms and delaying the worsening in RR-MS, fingolimod has very limited effect on the progressive forms of MS (Farez and Correale, 2016). For this reason, various selective S1P receptor modulators are currently under investigation in preclinical studies and in some cases in clinical trials to test their clinical use in primary or secondary progressive MS (Mao-Draayer et al., 2017).

Seeking for valuable alternatives to fingolimod, new-generation S1P receptor modulators have been developed, characterized by a higher selectivity and improved pharmacokinetic performance and tolerability (Pyne et al., 2016). A number of S1P receptor drugs in clinical trials of MS are reported in **Table 2** (Pyne et al., 2016; O’sullivan and Dev, 2017). Fingolimod is used in MS; however, ongoing preclinical studies suggest the use of fingolimod for different brain diseases, including AD, HD, and PD. In **Table 3**, fingolimod current clinical trials related to other brain diseases other than MS are summarized (O’sullivan and Dev, 2017).

**Table 2:** Use of S1PR drugs in clinical trial for central nervous system (CNS) disease.

S1PR drugs in clinical trial	Target	Indication for diseases
Fingolimod- FTY720	S1P_1_–S1P_5_	RR-MS
Siponimod- BAF312	S1P_1_, S1P_5_	SP-MS RR-MS
Ozanimod- RPC1063	S1P_1_	RR-MS
Ceralifimod- ONO-4641	S1P_1_, S1P_5_	RR-MS
GSK2018682	S1P_1_	RR-MS
Ponesimod- ACT-128800	S1P_1_	RR-MS
KRP203	S1P_1_	Ulcerative colitis Systemic lupus erythematosus
Cenerimod- ACT-33441	S1P_1_	Systemic lupus erythematosus
Amiselimod- MT1303	S1P_1_, S1P_4_, S1P_5_	RR-MS Crohn’s disease Psoriasis
Etrasimod- APD334	S1P_1_, S1P_4_, S1P_5_	Ulcerative colitis

RR-MS, relapsing–remitting multiple sclerosis; SP-MS, secondary progressive multiple sclerosis.

**Table 3:** Research and clinical use of FTY720/fingolimod.

Disease	Clinical trial	*In vitro* (reference)	*In vivo* (reference)
Amyotrophic lateral sclerosis	Phase II	Munoz-Saez et al., 2015	Potenza et al., 2016
Acute stroke	Phase II	Agudo-Lopez et al., 2010; Czubowicz et al., 2015	Czech et al., 2009; Shichita et al., 2009; Zhou et al., 2010; Liesz et al., 2011; Wei et al., 2011; Wacker et al., 2012; Yung et al., 2012; Liu et al., 2013a; Ichijo et al., 2015; Zheng et al., 2015
Schizophrenia	Phase II		Jang et al., 2011; Muhle et al., 2013
Rett syndrome	Phase I	Deogracias et al., 2012	Guy et al., 2001
Glioblastoma	Phase I	Paugh et al., 2009; Estrada-Bernal et al., 2012	Yoshida et al., 2010; Zhang et al., 2015
Alzheimer’s disease		Puglielli et al., 2003	Asle-Rousta et al., 2013a; Hemmati et al., 2013; Asle-Rousta et al., 2014
Huntington’s disease		Di Pardo et al., 2014; Miguez et al., 2015	Di Pardo et al., 2014
Parkinson’s disease		Pyszko and Strosznajder, 2014b; Vargas-Medrano et al., 2014	Komnig et al., 2018
Traumatic brain injury			Zhang et al., 2007; Zhang et al., 2008; Mencl et al., 2014; Novgorodov et al., 2014
Epilepsy			Hodgson et al., 1999; MacLennan et al., 2001; Mikati et al., 2003; Akahoshi et al., 2011; Gao et al., 2012
Pain		Chen et al., 2014	Coste et al., 2008; Doyle et al., 2011; Mair et al., 2011; Zhang et al., 2015
Krabbe’s disease		O’sullivan and Dev, 2015	Contreras et al., 2010

The role played by S1P in the development of neurodegenerative diseases like MS makes S1P receptors the most interesting pharmacological targets.

As mentioned above, the first molecule used as therapeutic agent for MS is fingolimod, which acts as an S1P_1,3,4,5_ modulator (Huwiler and Zangemeister-Wittke, 2018). Fingolimod, which in 2010 has been approved by the FDA for the treatment of RR-MS, is a prodrug whose chemical structure is very similar to that of sphingosine. Its phosphorylation turns it into (S)-FTY720-monophosphate, an S1P analog (Sushil and Batra, 2012). This phosphate-derivative undergoes the action of the same phosphatases (SPP1 and SPP2) that dephosphorylate S1P. To overcome this problem and in the wake of fingolimod’s success, structure-activity relationship (SAR) studies performed on FTY720-phosphate have been used to design several fingolimod-phosphate derivatives and nonhydrolyzable phosphonate (reviewed in Marciniak et al., 2018, and Stepanovska and Huwiler, 2019).

The key features of the agonists obtained after these modifications can be summarized as follows: polar head, aromatic region, and lipophilic tail (Liu et al., 2019). Indeed, not only molecules strictly similar to S1P are able to interact with S1P receptors.

**Figure 5:**
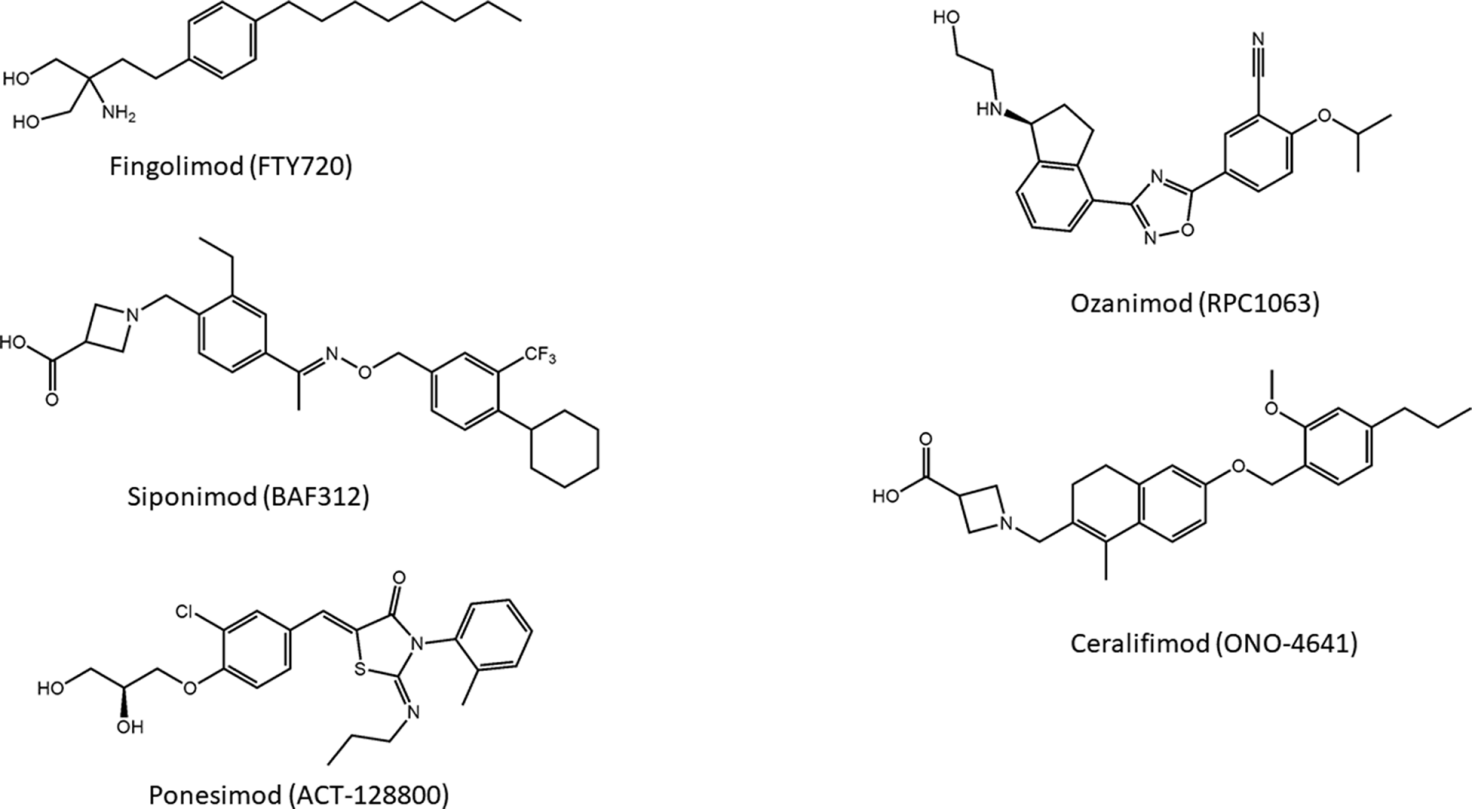
Novel modulators of S1P receptors.

Introduction of a carboxylic acid head group that does not require activation for bioavailability led to the development of a compound designated as BAF312, commercially known as siponimod (**Figure 5**), which entered phase III clinical studies on secondary progressive MS (SPMS) patients. This molecule displays potent agonism on S1P_1_ and S1P_5_ while completely sparing S1P_3_, and the highest selectivity toward S1P_1_ and S1P_5_ is caused by the increased rigidity in the lipophilic alkyl chain of fingolimod (Pan et al., 2013).

Another S1PR modulator and MS drug candidate is ONO-4641, commercially named ceralifimod (**Figure 5**). This molecule has a chemical structure similar to that of siponimod and also acts specifically on S1P_1_ and S1P_5_. A phase II clinical trial was concluded in 2012; however, at the moment, it is not clear what future developments the molecule will have (Subei and Cohen, 2015).

About the series of oxadiazole-based S1P agonists with high selectivity for S1P_1_ and S1P_5_ receptor, the derivative disubstituted on terminal benzene ring and with a terminal hydroxyl group RPC1063, named ozanimod (**Figure 5**) (Scott et al., 2016), has entered phase III clinical trials. Ozanimod, one of the most promising molecules for MS, has minor adverse effects and shows superior receptor specificity if compared to fingolimod, siponimod, and ponesimod (Westad et al., 2017).

Ponesimod (ACT-128800) (**Figure 5**) is another class of S1P_1_ receptor agonists based on the 2-imino-thiazolidin-4-one scaffold, with a diol substituent that does not require phosphorylation for the acidity. This compound has been selected for clinical development (Bolli et al., 2010), and its selectivity for S1P_1_ is approximately 650-fold higher compared with the natural ligand S1P (D’ambrosio et al., 2016).

Several other molecules have been developed and entered in phase I clinical trials, but other studies are needed to design more potent and selective immunomodulatory drugs without causing major adverse effects.

## Conclusions

Deregulation of sphingolipid metabolism is a common feature of a number of brain diseases, even in the absence of specific known defects of sphingolipid metabolic enzymes and/or sphingolipid intracellular traffic (Piccinini et al., 2010). Thus, researchers in this field have frequently explored the concept of targeting sphingolipid metabolism to address brain diseases. However, the translation of basic research to therapeutic opportunities in the case of complex glycosphingolipids has been so far unsuccessful. The only notable exception is represented by the potential use of a glucosylceramide synthase inhibitor, GZ667161, for the treatment of PD patients associated with mutations of the gene encoding for the glucocerebrosidase (Sardi et al., 2017). Dysregulation of S1P signaling and of the metabolic machinery involved in the control of S1P levels has been largely demonstrated in neuroinflammatory and neurodegenerative diseases. The translational research in the field of S1P has been definitely more successful; in particular, addressing the family of S1P receptors as therapeutic target has led to the introduction of fingolimod for the therapy of RR-MS. Fingolimod is recognized as a very efficient drug in RR-MS, and pharmacological research in this sense has continued, leading to analogues with improved pharmacokinetic features and with a potential usefulness in the treatment of other forms of MS, where the neuroinflammation still is a major player. The complexity of fingolimod action in the brain suggests that this or similar drugs might be useful to treat CNS illnesses other than MS. Since discovery of fingolimod, new-generation S1P receptor drugs are also being developed to target more specific S1P receptors. Overall, the family of S1PRs thus appears worthy of continued study and may provide significant therapeutic opportunities. Not only S1P receptors but also many of the enzymes involved in S1P metabolism bear the potential of promising therapeutic targets. From this point of view, in addition to the sphingosine kinases, the enzymes involved in the control of S1P levels along the catabolic pathways have been recently emerged as crucial in mediating the pathological role of S1P in brain disease. As an example, it is worth to remind that mutations in the S1P lyase are associated with neural toxicity (Choi and Saba, 2019). Thus, the array of possible druggable targets is considerably widening, and we would expect exciting developments in this field in the near future.

## Author Contributions

AP and PG contributed to the conception and design of the paper. AP wrote the first draft of the paper. AP, SG, LM, SP, and PG wrote sections of the paper. LC designed the graphics. All authors contributed to critical analysis of the literature, contributed to manuscript revision, and read and approved the submitted version. SG and LM equally contributed to the paper, and their names are listed in alphabetical order.

## Conflict of Interest Statement

The authors declare that the research was conducted in the absence of any commercial or financial relationships that could be construed as a potential conflict of interest.

## Abbreviations

3-kdhSo, 3-ketodihydrosphingosine; AD, Alzheimer’s disease; APP, amyloid precursor protein; BBB, blood brain barrier; CerS, ceramide synthases; CDase, ceramidase; CNS, central nervous system; DES1, dihydroceramide desaturase 1; dhCer, dihydroceramide; dhSo, dihydrosphingosine; EAE, experimental autoimmune encephalomyelitis; GCase, glycosidases; HD, Huntington disease; HDAC(s), histone deacetylase(s); KDSR, 3-ketodihydrosphingosine reductase; LPS, lipopolysaccharide; MS, multiple sclerosis; NF-κB, nuclear factor κB; PD, Parkinson’s disease; PI3K, phosphoinositide 3-kinase; PS1, presenilin-1; ROS, reactive oxygen species; S1P, sphingosine 1-phosphate; S1P_1-5_, S1P receptor 1 to 5; SGPL1, sphingosine 1-phosphate lyase; SK1, sphingosine kinase 1; SK2, sphingosine kinase 2; SM, sphingomyelin; SMase, sphingomyelinase; SPP1, S1P phosphatase; SPT, serine palmitoyltransferase; RR-MS, relapsing–remitting multiple sclerosis; SP-MS, secondary progressive multiple sclerosis.
